# Research Progress on Dance Training as a Mechanical Stimulus for the Prevention and Treatment of Osteoporosis: A Narrative Review

**DOI:** 10.3390/ijms27052185

**Published:** 2026-02-26

**Authors:** Yunli Jia, Fan Yu, Wei Wu

**Affiliations:** 1 School of Dance, Northwest Minzu University, Lanzhou 730030, China; 2 School of Exercise and Health, Shanghai University of Sport, Shanghai 200438, China; 3 School of Athletic Performance, Shanghai University of Sport, Shanghai 200438, China

**Keywords:** osteoblast, bone, bone mineral density, dance, signaling pathway

## Abstract

From a mechanical stimulation perspective, this study aims to explore the mechanisms by which dance training affects bone metabolism and to clarify its potential as a preventive and therapeutic measure for osteoporosis (OP). A comprehensive search was conducted on the PubMed, Web of Science, and China National Knowledge Infrastructure databases, utilizing search terms related to dance, mechanical stimulation, and OP. The present study incorporated a wide range of research methodologies, including randomized controlled trials, observational studies, systematic reviews, and narrative reviews, with the overarching objective of encompassing all pertinent concepts within the purview of our investigation. The synthesis and description of the search results were conducted through a narrative approach. A preliminary investigation of extant literature suggests that studies that comprehensively delineate the mechanism of action between dance and OP are few and far between. However, a thorough review of the extant literature indicates that dance training can enhance bone density and promote skeletal health by influencing mechanical characteristics. Concurrently, dance can function as a mechanical stimulus, thereby regulating bone metabolism by activating relevant cellular signaling pathways, thus contributing to the reduction of bone loss. Dance training, as a form of mechanical stimulation, has the potential to play a crucial role in the prevention and treatment of OP. However, the precise nature of the dance styles, the training intensities and frequencies remains unclear. It is recommended that future research endeavors concentrate on the comprehensive integration of these measures, with the objective of addressing the existing knowledge gaps. This approach is expected to provide a more robust evidence base for the utilization of dance-based strategies in the prevention and management of OP.

## 1. Introduction

Osteoporosis (OP) is a prevalent metabolic bone disorder characterized by reduced bone mass and impaired bone microarchitecture and is accompanied by an increased risk of fragility fractures, particularly among the aging population [1]. The etiology of OP is multifactorial, with potential genetic components, the effects of aging, endocrine disorders, malnutrition, and adverse lifestyle habits such as smoking and alcohol abuse all contributing to its development [1]. According to the latest statistics, approximately 8.9 million people worldwide suffer fractures due to OP annually, with the annual fracture risk for patients increasing from 60% to 82%, imposing a significant burden on human health and socioeconomic systems [2]. Currently, pharmacotherapy remains the primary treatment for OP. Conventional anti-OP therapies include antiresorptive agents and anabolic agents [1]. While these pharmaceutical interventions can effectively regulate the progression of OP, concerns regarding long-term medication use persist. These include variations in efficacy, substantial adverse effects, and a substantial financial burden [2]. Exercise is recognized as one of the most prevalent, dependable, and economical interventions for the prevention and treatment of OP. The World Health Organization has classified exercise as the nonpharmaceutical physical therapy of choice for the management of OP (e.g., moderate-intensity treadmill exercise, high-intensity resistance training combined with impact training, etc.) [3,4,5,6]. Dance has gained global popularity as a form of physical activity [7]. Its benefits, which include flexibility in terms of time and location, safety, and ease of learning, have made it an effective measure for preventing OP intervention, such as aerobic dance and recreational dance [8,9,10,11,12,13,14,15]. Therefore, this study explored the effects and potential mechanisms of dance training as a mechanical stimulus and its biomechanical effects on bone mineral density (BMD) and related bone metabolism in OP patients, in order to provide new theoretical support for OP prevention and treatment strategies.

## 2. Materials and Methods

This narrative review employed a comprehensive keyword search strategy to retrieve studies from the PubMed, Web of Science, and China National Knowledge Infrastructure databases, covering the period from inception to October 2025. The search terms employed in this study included “dance”, “dance training”, “mechanical stimulus”, “osteoporosis”, “bone”, “bone mineral density”, “osteoblast”, and “osteoclast”.

A comprehensive selection of relevant studies was made, including reviews, original research, online reports, and e-books, to provide an overview of the most pertinent information on this topic. The research agenda was structured around two key pillars: original research and systematic reviews. Original research was prioritized for its direct relevance to the conceptual framework of this study, while systematic reviews were synthesized to provide comprehensive evidence syntheses. In instances where two studies reported analogous information, the more recent and exhaustive study was incorporated into the manuscript. In the course of this review, commentaries, correspondence submitted for publication, protocols, and trial registries were excluded from consideration. While not exhaustive, this review synthesizes key findings from the literature to provide the most comprehensive overview possible of the effects of dance training on OP.

## 3. An Examination of Dance in General

Dance is an art form that employs refined, organized, and artistically crafted human movement as its primary means of expression. The integration of elements such as music, costumes, lighting, and stage design enables the conveyance of ideas and emotions while simultaneously portraying social life within defined spatial and temporal contexts [16]. The significant dance classifications include stage performance, social dance, traditional dance, and popular dance. The fundamental components of movement include force, speed, amplitude, and direction [17]. While the exercise intensity of dance is significantly lower than that of sports, such as soccer or track and field, some sources indicate that dance surpasses badminton in intensity and ranks just below tennis [18]. These actions can deliver stress stimuli to bones through relatively high-intensity exercise, thereby increasing bone strength and BMD [18].

A substantial body of research has validated the efficacy of dance as a form of exercise intervention, demonstrating its positive impact on BMD and bone strength across diverse populations, thereby effectively mitigating the development and progression of OP. Zhou et al. [19] enrolled 28 postmenopausal women in a dance intervention program consisting of five 60-min sessions per week for a period of six months. The results revealed significant improvements in the participants’ lipid profiles, hormonal status, and immune function following six months of dance training, accompanied by substantial increases in BMD at the lumbar spine (L2-L4), greater trochanter, and femoral neck. Bao et al. [20] conducted a study comparing elderly women with more than two years of professional dance training as the experimental group against elderly women who rarely engaged in effective physical activity as the control group. The investigation revealed that elderly women with extensive long-term dance training presented significantly greater bone strength than did the control group, further substantiating the positive impact of dance on BMD. A similar study of underweight female college students found that after 18 weeks of dance instruction and training, BMD increased significantly at the third and fourth lumbar vertebrae, the hip, the femur, and the calcaneus. These findings suggest that dance training has a positive effect on BMD in this population [21]. In addition, when incorporated into a structured dance training regimen, aerobic dance has been shown to normalize serum levels of bone turnover biomarkers that are often dysregulated in conditions such as OP. These biomarkers include osteocalcin (OCN) and alkaline phosphatase (ALP), both of which are essential for normal bone formation and remodeling. While elevated serum concentrations of OCN and ALP are frequently observed in postmenopausal women and reflect dysregulated bone metabolism, these molecules themselves are not detrimental to bone tissue function [22]. Instead, their abnormal levels serve as indicators of underlying pathological processes, and aerobic dance may help restore their physiological ranges. Furthermore, aerobic dance has been shown to influence calcium metabolism balance through cardiovascular adaptation and endocrine regulatory mechanisms. This provides essential minerals for bone formation, thereby increasing local or systemic bone mass [23].

In summary, dance training plays a positive role in preventing OP and delaying its progression by enhancing BMD and bone strength.

## 4. Effect of Mechanical Stimuli on Skeletal Adaptation—The Mechanosensitive Cell Populations

Bone is not an immutable, static structure; rather, it is a dynamic tissue that is in a constant state of bone remodeling. Bone remodeling is a dynamic, continuous process that is essential for maintaining skeletal integrity and mineral homeostasis [24]. The remodeling cycle comprises three consecutive phases: first, the resorption phase, in which osteoclasts (OCs) degrade old bone tissue; second, the reversal phase, during which mesenchymal-derived osteoblasts (OBs) are recruited to the sites of bone resorption; and third, the formation phase, in which OBs synthesize and deposit new bone until the resorbed old bone is completely replaced [24]. During the process of bone remodeling, OBs and OCs function as primary cells and play pivotal roles [24]. Disruption of the remodeling cycle or an imbalance between resorption and formation due to cyclical abnormalities can induce metabolic bone diseases, with OP being the most prevalent among them [25]. Wolf’s law, established in the 19th century, posits that bones respond to sustained external stress by increasing bone hardness through adjustments in BMD and bone architecture. Conversely, a state of minimal stress has been shown to induce processes of resorption and weakening, which collectively result in bone loss [26]. For example, high-impact, load-induced stresses from activities such as weightlifting and jumping have been shown to promote bone formation and help prevent resorption during remodeling [26]. Conversely, prolonged disuse or mechanical unloading—such as extended bed rest or spaceflight—has been shown to accelerate bone apoptosis, leading to bone loss and conditions such as disuse OP [27].

During the effector cell response phase, the skeletal impact of mechanical loading is governed by the magnitude, duration, and loading rate of the applied stimulus [28]. Evidence suggests that loading protocols characterized by longer durations and lower amplitudes can elicit osteogenic responses comparable to those of shorter duration and higher amplitude [28]. Notably, while static loading in vivo fails to induce bone formation, dynamic mechanical loading is a potent osteogenic stimulus, particularly when peak strain exceeds approximately 1000 µstrain. However, when strain levels surpass the threshold of 3000 ustrain, micro-damage accumulates rapidly, potentially compromising bone tissue integrity and homeostasis [28]. Ignatius et al. [29] demonstrated that uniaxial cyclic strain (1%, 1 Hz, 1800 cycles/day for 3 weeks) significantly enhances the proliferation, differentiation, and osteogenic gene expression of human fetal OBs (hFOB 1.19). Conversely, sustained compressive loading (0–10.0 g/cm2 for 48 h) has been shown to stimulate osteoprotegerin (OPG) production in murine OBs via the non-canonical Wnt/Ca^2+^ signaling pathway. This elevation in OPG levels inhibits osteoclastogenesis by competitively blocking the RANK/RANKL interaction [30]. Furthermore, Wehrle et al. [31] established that periodic mechanical strain (8–16 N, 10 Hz, 3000 cycles; 3 times/week for 4 weeks) significantly accelerates callus formation and mineralization in murine fracture models. This regenerative effect is likely mediated by the activation of the Wnt signaling pathway and the subsequent downregulation of sclerostin (SOST) and RANKL distribution within the fracture callus.

The microscopic basis for this macroscopic phenomenon is mechanosignaling. Osteocytes, the most abundant cells and primary mechanical sensors in bone, detect mechanical loads through their cell bodies, dendritic processes, and specialized primary cilia. These cilia perceive fluid flow and convert shear stress into biochemical response signals [32]. While this cellular activity exerts a substantial influence on the skeletal system [33,34], the precise mechanisms by which osteocytes coordinate adaptive changes in bone mass and structure remain incompletely understood. It is widely accepted that when bones bear mechanical loads, this drives interstitial fluid flow within the unmineralized periosteal matrix surrounding osteocytes and their dendritic projections [35]. This flow activates OBs through as yet unidentified pathways, prompting them to produce signaling molecules—including Wnts, prostaglandin E2 (PGE2), osteomorphin, and nitric oxide—that regulate the activity, differentiation, and recruitment of OBs and OCs. This process is instrumental in preserving bone mass stability and structural integrity, orchestrating the process of bone remodeling [36]. Furthermore, Tatsumi et al. [37] reported that following selective depletion of osteocytes in mice, the animals exhibited a classic brittle bone phenotype, including internal cortical bone porosity, microfracture formation, and other characteristic alterations associated with aging bone tissue. Notably, these mice with osteocyte dysfunction exhibited remarkable resistance to mechanical unloading-induced bone loss. This finding directly corroborates the critical role of osteocytes in mechanical signal transduction. Consequently, any form of exercise that provides effective mechanical stimulation—including dance training—has the potential to serve as a powerful tool for promoting bone health.

## 5. Mechanical Stimulation Activates Key Cellular Signaling Pathways

Dance training has been demonstrated to be a form of mechanical stimulation that influences the prevention and treatment of OP by activating several cellular signaling pathways. As a form of mechanical stimulation, dance training may play a significant role in the prevention and treatment of OP by activating multiple cellular signaling pathways.

### 5.1. Integrins

Integrins are transmembrane adhesion receptors located on the cell surface that function as sensors, detecting mechanical changes in the extracellular matrix (ECM). They play pivotal roles in mechanical signal transduction pathways [38]. Integrin-associated core mechanosensitive proteins constitute a vital component of the mechanical signaling pathway and include members such as focal adhesion kinase (FAK), c-Src, vinculin, talin, F-actin, and other associated proteins. These proteins receive signals transmitted by integrins and transduce them into the cytoplasm, triggering signal cascades associated with terminal cellular responses [38]. As secreted extracellular matrix components, CCN family proteins play key regulatory roles in bone tissue development and remodeling [38]. Among them, CCN3 has been shown to bind specifically to integrin α5β1, inducing the phosphorylation and activation of FAK and Akt. This, in turn, results in the upregulation of the expression levels of the key osteogenic transcription factors Runx2 and Osterix [39]. Additionally, in osteocytes, the integrin-mediated signaling pathway is indispensable for the mechanical stress-induced osteogenic response. Integrins, as upstream components of signal transduction, sense mechanical stress signals and activate FAK, thereby initiating the intracellular transmission of mechanical signals [40]. Hu et al. [41] demonstrated that the silencing of FAK in bone marrow mesenchymal stromal cells (BMSCs) led to significant suppression of the uniaxial mechanical stretching-induced osteogenic differentiation of BMSCs. This suppression was accompanied by a reduction in ALP and Runx2 expression, as well as an inhibition of the activation of related downstream proteins. This finding further substantiates the crucial role of integrin-mediated mechanical signaling perception in tension-induced osteogenesis. Further studies have indicated that the elimination of integrin-β1 impedes the capacity of mechanical strain to effectively activate the β-catenin signaling pathway while concomitantly exerting a substantial inhibitory effect on OB differentiation. This finding indicates that the promotion of OB differentiation by mechanical strain is contingent upon integrin-β1-mediated β-catenin signaling, thereby augmenting the responsiveness of bone tissue to mechanical loading [42]. Therefore, integrins and their downstream pathways function as critical conduits, effectively converting mechanical stress into osteocytic responses. These pathways play pivotal roles in the process of mechanical stimulation, which is essential for counteracting OP.

**Figure 1:**
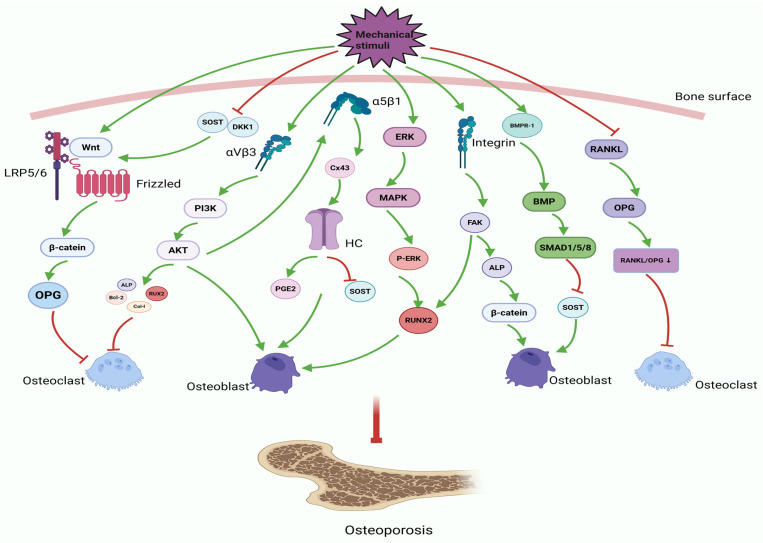
Mechanical stimulation activates cellular signaling pathways for OP prevention and treatment. Mechanical stimulation exerts a bone-protective effect by activating the Wnt/β-catenin pathway, PI3K/Akt/mTOR signaling pathway, TGF-β/BMP/SMAD pathway, and ERK/MAPK signaling pathway, promoting integrin expression, and reducing the RANKL/OPG ratio.

Furthermore, studies have revealed that the hedgehog [43] signaling pathway interacts with the Wnt and BMP signaling pathways to coordinate skeletal growth [44]. The Indian hedgehog (Ihh) is a pivotal factor in the process of endochondral ossification, in which the activation of Hh signaling plays a crucial role in regulating the synchronized progression of both chondrogenesis and osteogenesis. This is achieved by influencing the differentiation and proliferation of chondrocytes, as well as the directed differentiation of OBs [45]. Mechanical stimulation activates the Hh signaling pathway, inducing OB proliferation through Hh signaling mediation [46,47]. This finding indicates that the Hh pathway may have a regulatory role in bone density and could serve as a therapeutic target for the treatment of OP [44]. In essence, there is regular mechanical loading on the skeletal system and several cellular pathways, but the output is improved bone health. As illustrated in Figure 1, the system comprises a number of components and pathways, yet numerous connections and interactions remain to be elucidated. It is recommended that future research endeavors direct their attention towards a more comprehensive investigation of the underlying mechanisms of action.

### 5.2. ERK/MAPK Signaling Pathway

Extracellular signal-regulated kinase (ERK), an evolutionarily ancient signal transducer within the mitogen-activated protein kinase (MAPK) family, has long been implicated in regulating OB differentiation and bone formation [48]. ERK/MAPK signaling has been demonstrated to be essential for the differentiation of human OBs and bone marrow stromal cells [49]. For example, the ERK/MAPK pathway has been shown to induce the activation of ATF4, RUNX2, and β-catenin in OBs. In addition, mice lacking Erk1, Erk2, Mek1, or Mek2 exhibit severe bone loss, limb deformities, and impaired skeletal mineralization [50]. Mechanical stimuli, such as loading in the form of fluid flow stress, have been shown to induce a series of rapid responses within OBs by activating the ERK/MAPK pathway [51]. Upon activation by mechanical stimuli, the ERK/MAPK pathway induces the production of the active form, P-ERK. The active molecule subsequently translocates to the nucleus, where it binds specifically to Runx2, which is prebound to the chromatin of osteogenic target genes (e.g., Bglap2, Ibsp). Runx2 is subsequently phosphorylated at sites S301 and S319, thus activating its transcriptional function. This process ultimately regulates the expression of downstream osteogenic-related genes [52]. Research has confirmed that oscillatory fluid flow stimulates the ERK1/2 pathway within osteocytes, synergistically regulating their secretory phenotype alongside the Wnt/β-catenin pathway. Activated osteocytes effectively suppress OC generation and activity while promoting OB differentiation to maintain bone remodeling equilibrium [51]. Consequently, mechanical stimulation has been demonstrated to promote OB function and indirectly suppress OC activity through the ERK/MAPK pathway, thereby contributing to bone mass maintenance.

### 5.3. Wnt/β-Catenin Signaling Pathway

The Wnt/β-catenin pathway is a well-established regulator of bone formation and plays a crucial role in maintaining bone mass [53]. The process of Wnt signaling involves the binding of Wnt to low-density lipoprotein receptor-related protein 5/6 (LRP5/6) and Frizzled receptors. This binding activates β-catenin, which plays a crucial role in the activation of downstream pathways. This process has been shown to promote the differentiation of OBs and stimulate bone matrix synthesis, thereby contributing to the regeneration and repair of the entire bone tissue [54]. Mechanical stimulation has been demonstrated to activate the Wnt/β-catenin pathway. For example, mechanical stress influences bone tissue signaling pathways through two mechanisms. First, it directly activates the Wnt/β-catenin pathway by upregulating the expression of Wnt ligands, their receptors, β-catenin, and downstream transcription factors while downregulating the expression of Wnt pathway inhibitors, ultimately promoting bone formation. On the other hand, it can also indirectly activate the Wnt/β-catenin pathway by regulating metabolic levels at the tissue and cellular levels through physiological stimulation [55]. Furthermore, mechanical stress has been demonstrated to downregulate the secretion of inhibitory factors, such as SOST and Dickkopf-related protein 1 (DKK1), by osteocytes, thereby relieving the inhibition of Wnt signaling [56]. Enhanced Wnt signaling has been shown to stimulate OBs and endothelial cells to produce more OPG, thereby reducing the receptor activator of NF-κB ligand (RANKL)/OPG ratio. This decrease has been shown to inhibit OC generation and bone resorption while suppressing OC differentiation [57]. Research has demonstrated that mutations in Wnt1 result in a reduction in β-catenin levels, thereby diminishing Wnt/β-catenin signaling and ultimately leading to OP [58]. Consequently, mechanical stimulation has been demonstrated to promote a restorative balance between bone formation and resorption by activating the Wnt/β-catenin pathway. This finding has significant implications for the prevention and treatment of OP.

### 5.4. PI3K/Akt/mTOR Signaling Pathway

The phosphoinositide 3-kinase (PI3K)/protein kinase B (AKT)/mammalian target of rapamycin (mTOR) signaling pathway is one of the most critical signaling pathways in cells. This pathway is involved in the regulation of bone metabolism and contributes to the proliferation and differentiation of bone marrow mesenchymal stem cells, OC and OB. It plays a pivotal role in bone metabolism and bone remodeling [59]. As demonstrated in prior studies, activation of the PI3K/Akt signaling pathway protects OBs from tumor necrosis factor-α (TNF-α)-induced apoptosis and promotes apoptosis in OCs. Inhibition of this pathway has been shown to partially block its antiapoptotic effects [60]. PI3K can act as a key mediator in mechanotransduction [61]. Specifically, mechanical stress initially activates integrin αVβ3 on OB dendrites, functioning as a mechanical sensor, which subsequently initiates the PI3K/AKT signaling pathway. This pathway subsequently activates integrin α5β1 at the cell body, which, upon stimulation, binds to connexin 43 (Cx43), thereby inducing the opening of hemichannels (HCs). Once HCs are opened, they not only release molecules that promote bone synthesis, such as prostaglandins, but also downregulate the expression level of the bone formation inhibitor SOST. This ultimately achieves a bone-promoting effect [61]. Furthermore, the PI3K/Akt/mTOR signaling pathway has been shown to convert mechanical signals into biochemical signals, thereby regulating cell morphology, the expression of osteogenic markers ALP, runt-related transcription factor 2 (Runx2), and collagen I (COL-I), and the differentiation fate toward the osteogenic lineage [62]. Moderate activation of this pathway has been shown to inhibit OB apoptosis by activating prosurvival proteins, including members of the Bcl-2 family. This process contributes to the maintenance of bone homeostasis. Inhibiting this pathway has been shown to downregulate p-Bad expression and partially block the antiapoptotic effects of OB, ultimately increasing the Bax/Bcl-2 ratio and activating caspase-3 [63]. Consequently, mechanical stimulation-activated PI3K/Akt/mTOR signaling exerts a bone-protective effect, achieving therapeutic benefits for OP.

### 5.5. TGF-β/BMP/SMAD Signaling Pathway

Transforming growth factor-β (TGF-β) and its superfamily member bone morphogenetic proteins (BMPs) play crucial roles in OB and chondrocyte lineage differentiation, skeletal development, and homeostasis [64]. BMP signaling has been demonstrated to induce the expression of multiple osteogenesis-related transcription factors (Runx2, forkhead box C1 (Foxc1), msh-homebox 2 (Msx2), etc.). In addition to this effect, BMP signaling positively regulates mTORC1, thereby promoting OB activity [64]. The suppressor of the mother against decapentaplegic (SMAD) represents the most critical downstream signaling mediator of TGF-β/BMP intracellular signaling. The activation of SMAD 1/5/8 has been shown to promote the upregulation of osteogenic gene expression [65]. Research has demonstrated that mechanical loading exerts a regulatory influence on bone metabolism, specifically by stimulating the BMP/TGF-β/SMAD signaling pathway [66]. The biomechanical stimulation of OBs activates SMAD1/5/8 in the BMP signaling pathway via BMPR1, enhancing osteogenesis by upregulating SMAD-dependent osteogenic genes [65]. Nguyen et al. [67] found that when axial compressive loads equivalent to 10 times the body weight were applied to mice, this load suppressed the phosphorylation process of the TGF-β effector molecule SMAD3. This, in turn, diminished the functional activity of SMAD3 within osteocytes. Concurrently, this intervention blocked the SMAD3-induced upregulation of SOST gene expression, ultimately manifesting as reduced osteosclerostin secretion and increased bone mass. Furthermore, mechanical stimulation has been demonstrated to induce the secretion of BMP-7 by OBs. The binding of BMP-7 to its receptor, BMPR2, has been shown to activate the PI3K/AKT/GSK3β pathway, thereby inhibiting glucocorticoid-induced OB apoptosis (e.g., dexamethasone) [68]. Consequently, mechanical stimulation has been demonstrated to modulate bone formation by activating the TGF-β/BMP/SMAD signaling pathway.

### 5.6. RANKL/RANK/OPG Signaling Pathway

The receptor activator of nuclear factor kappa B (RANKL)/receptor activator of nuclear factor kappa B [69]/osteoprotegerin (OPG) signaling pathway is a core pathway regulating OC differentiation and function [70]. RANKL, a homotrimeric transmembrane protein belonging to the TNF superfamily, is produced by osteocytes and other cells. This process stimulates and maintains the differentiation of pre-OCs [71]. The expression of RANK is catalyzed by OCs and their precursors [72]. In fact, RANKL, secreted by osteocytes and OBs, binds to the RANK receptor expressed on the surface of OCs. This interaction not only promotes the differentiation of OCs but also accelerates the generation of mature OCs. In contrast, OPG functions as a decoy receptor for RANKL, competitively binding to RANKL and thereby impeding its interaction with RANK. Consequently, increasing the OPG/RANKL ratio has been shown to inhibit OC formation [71]. Mechanical stress exerts a primary regulatory influence on bone remodeling by affecting the RANKL/OPG balance. Moderate mechanical loading has been demonstrated to reduce RANKL expression in bone tissue while increasing OPG expression. Consequently, this results in a decrease in the RANKL/OPG ratio and subsequent suppression of OC activity [71]. For example, treadmill exercise and vibration stimulation reduce RANKL expression and increase OPG expression in glucocorticoid-induced osteoporotic rats, thereby inhibiting RANKL and RANKL-induced bone loss [73]. Conversely, in the absence of mechanical stimulation, the RANKL/OPG ratio increases, promoting OC activity and bone loss. For example, in rats with disuse OP, which was induced by tail suspension (lacking mechanical loading), the expression levels of genes associated with OC differentiation—such as RANKL, RANK, IL-6, and TNF-α—were significantly greater than those in normal controls, whereas the expression level of OPG, a factor associated with OC differentiation inhibition and formation, was markedly lower than that in normal controls. These findings indicate that the absence of mechanical loading can promote OC differentiation by regulating the expression of cytokines related to the RANKL/RANK/OPG system in the bone marrow microenvironment [74]. Consequently, the RANKL/RANK/OPG pathway has emerged as a pivotal mechanism through which mechanical stress modulates bone resorption. Mechanical stimulation has been demonstrated to inhibit OC activity through this pathway, thereby playing a crucial role in counteracting OP.

In summary, mechanical stimulation maintains bone homeostasis and counteracts OP by modulating integrins, the ERK/MAPK signaling pathway, the Wnt/β-catenin signaling pathway, the PI3K/Akt/mTOR signaling pathway, the TGF-β/BMP/SMAD signaling pathway, and the RANKL/RANK/OPG signaling axis. These pathways do not operate in isolation but instead form an integrated network that orchestrates bone remodeling. Integrins function as primary mechanosensors that initiate downstream signaling cascades, with FAK/Src-mediated activation of PI3K/Akt and ERK/MAPK pathways acting as key early events [38]. These cytoplasmic signaling cascades converge with the Wnt/β-catenin pathway, which is essential for osteogenesis. This convergence is frequently facilitated by the mechanical suppression of osteocyte-derived inhibitory factors such as SOST [75]. Mechanical loading not only enhances Wnt signaling by downregulating SOST levels but also modulates the TGF-β/SMAD pathway, thereby further suppressing SOST expression and amplifying pro-osteogenic signals [67]. Ultimately, this cross-regulatory network converges on core transcriptional regulators such as Runx2 to modulate the OPG/RANKL ratio and inhibit osteoclastogenesis [76,77]. The integrated mechanism ensures that diverse mechanical cues are efficiently transduced into a coordinated response that simultaneously promotes bone formation and inhibits bone resorption.

## 6. Mechanical Stimulation and Hormonal Responses

Beyond its direct mechanical effects on osteocytes via signaling pathways, dance training—acting as a form of mechanical stimulation—regulates bone metabolism by modulating the levels of osteogenic hormones and metabolic factors. The systemic response to moderate exercise alters hormonal profiles, thereby indirectly promoting bone health. Insulin-like growth factor-1 (IGF-1), primarily synthesized in the liver under the regulation of growth hormone (GH), mediates cellular differentiation, proliferation, and the expression of extracellular matrix proteins. It plays a pivotal role in bone remodeling and formation, and its levels are positively correlated with BMD [78]. Similarly, GH has been shown to facilitate skeletal development and remodeling by promoting the proliferation and function of chondrocytes, OBs, and OCs [79]. Research confirms that physical exertion triggers GH secretion [80]. Concurrently, exercise-induced mechanical loading benefits bone tissue by stimulating IGF-1 release from skeletal muscle [79]. Furthermore, BMD is strongly associated with estrogen levels [81]. Specifically, estrogen enhances TGF-β release from OBs, which inhibits OC activity and mitigates bone loss during resorption [82].

Dance training has been shown to enhance bone health by elevating systemic estrogen levels. For instance, Zhou et al. [19] reported that postmenopausal women engaging in a six-month dance intervention exhibited significantly higher serum levels of sex hormones, specifically estradiol (E2) and testosterone (T), compared to baseline. Accompanying these hormonal changes, significant increases in BMD were observed in the L2-L4 lumbar spine, femoral neck, and greater trochanter. Furthermore, Alfacalcidol (active vitamin D3) is known to prevent fractures in patients with OP by reducing plasma parathyroid hormone levels and inhibiting bone resorption [83]. Tan et al. [84] demonstrated that a combined intervention of dance exercise and alfacalcidol in middle-aged and elderly patients with OP resulted in significant improvements in BMD across multiple skeletal sites. This combination also notably enhanced patients’ activities of daily living and overall quality of life. In summary, the mechanical stimuli generated by dance training promote bone formation and attenuate bone resorption through metabolic mediators and hormones, such as IGF-1 and estrogen. This mechanism enables bone tissue to adapt to mechanical stress, thereby maintaining or augmenting bone mass and preserving skeletal health.

## 7. The Biomechanical Stimulation of Dance Training on OP

Research indicates that the structural adaptations induced by dance in the tibia are comparable to those observed in high-impact and repetitive impact activities. This suggests that various forms of dance can positively influence key skeletal parameters, such as bone mineral density and strength. These adaptive changes may help delay or prevent the onset of OP in later life [85]. The positive impact of dance training on the skeletal system may be due to its unique, highly effective biomechanical stimulus. This stimulus is analogous to the “master switch” that initiates self-repair and strengthening processes for bones. Therefore, to understand this mechanism, it is necessary to first examine the fundamental physiological characteristics and biomechanical principles of bones and then analyze how dance training promotes bone health [18,86,87].

### 7.1. Mechanical Characteristics of Dance Training

The positive effects of dance training on bone health have been attributed to the generation of multiple mechanical stimuli. These stimuli possess sufficient intensity and act upon the skeletal system in diverse forms and from all directions.

#### 7.1.1. Ground Reaction Force (GRF)

The GRF is a critical metric for evaluating the intensity of skeletal impact during movement. The GRF values have been utilized as a surrogate measure of the external gravitational loads experienced during various activities [88]. They are frequently employed in studies examining impact loads, athletic performance, human movement and gait patterns, as well as musculoskeletal pathologies [89]. In accordance with Newton’s Third Law of Motion, when dancers apply force to the ground, the ground exerts an equal and opposite force. This force is transmitted through the sole to the entire skeletal system, thereby serving as a key stimulus for the OB. The magnitude (peak value) and application rate (loading rate) of the GRF have been shown to jointly determine its osteogenic effects [90,91]. A substantial body of research has demonstrated the efficacy of impact-based activities characterized by high peak values and rapid loading rates in enhancing BMD [90,92,93,94]. For example, Ebben et al. [92] demonstrated that deep jumps generate the highest peak GRF and rate of force development (RFD) compared with jogging or walking, exerting a positive influence on BMD and osteogenic potential. Ng et al. [95] also demonstrated, through a systematic review and meta-analysis, that exercises generating GRF equal to or greater than those of running can significantly improve bone mineral density in the distal tibial cancellous bone of postmenopausal women. Furthermore, mechanical stimuli, such as GRFs, are converted into biochemical signals through mechanotransduction processes. These forces activate integrins and their downstream effectors, including the ERK, PI3K/Akt, and Wnt signaling pathways. This molecular cascade plays a pivotal role in modulating bone remodeling, thereby maintaining skeletal homeostasis, enhancing BMD, and mitigating the progression of OP [38,77]. However, it is imperative to acknowledge the limitations of this approach. The promotion of osteogenic effects by the peak GRF and loading rate has an upper limit. Once both parameters exceed the skeletal “adaptation threshold”, the osteogenic effect ceases to increase, and bone damage ensues.

A variety of dance movements, including jumps, steps, runs, and rapid shifts in the center of gravity, have the capacity to generate significant GRF. A biomechanical study of traditional dance revealed a correlation between the vertical GRF and loading rate and the dance experience level. Although professional dancers exhibit lower peak GRF than inexperienced individuals do, they maintain loading rates within a high-signal-efficiency, injury-free range through precise movement control (e.g., cushioning coordination upon landing). This approach activates osteogenesis while mitigating injury risks associated with high peak forces. In contrast, novice dancers have been shown to generate higher peak GRF and loading rates, resulting in disrupted osteogenic signaling and an increased risk of musculoskeletal injury [86]. Furthermore, tap dancers have been shown to generate relatively low GRF, joint forces, and joint moments, thereby reducing their risk of injury [96]. Farnell et al. [11] also demonstrated that the multiplanar GRF experienced by dancers during movement may be sufficient to increase lumbar spine BMD. However, Alfonso et al. [97] found that during high-frequency, high-impact flamenco steps without jump-cushioned movements, the GRF directly impacts joints (heel strikes exceeding twice body weight), increasing knee loading, and ultimately leading to knee injuries. Consequently, optimal GRF and loading rates are conducive to bone health, whereas excessive levels result in related injuries.

#### 7.1.2. Muscular Mechanics

Intricate interconnections between muscles and bones characterize the musculoskeletal system. During physical activity, the nervous system regulates and drives skeletal muscles, causing asynchronous sliding of actin and myosin filaments. This, in turn, triggers muscle contraction. This process generates a variety of movement torque patterns at joints, thereby enabling multidirectional bone movement [98]. Additionally, skeletal muscles are recognized as the predominant source of mechanical loading on bones, and the development and maintenance of bone mass are significantly influenced by mechanical stress from muscles [98]. Skeletal muscle and bone constitute an integrated organ system that is connected primarily through mechanical coupling. Skeletal structures serve as attachment points for muscles, and the mechanical loads generated by muscle contraction synchronously regulate bone remodeling and metabolic processes [99].

It has been demonstrated that dance training can serve as an effective stimulus for enhancing musculoskeletal adaptability, improving strength levels, and strengthening coordination of extension and flexion [100]. Xie et al. [18] confirmed that long-term dance training among female college students significantly enhances lumbar spine BMD, with the most pronounced effects observed at the L2 and L3 segments. The mechanism under discussion involves the stretching stimulation of the lumbar, abdominal, hip, and thigh muscles that is inherent to dance training. This stimulation has been demonstrated to promote muscle metabolism, increase muscle mass and strength, and thereby simultaneously amplify mechanical loading on bone tissue and improve BMD and bone strength. This training has been shown to assist female college students in optimizing peak bone mass, while concurrently preventing and improving bone loss during the postmenopausal period. Similarly, Zhou et al. [19] observed that dance movements, including hip rotations and waist extensions, offer a comprehensive exercise regimen for joints and muscles. The sustained exercise load and muscle stretching effectively increase OB activity, thereby supporting bone formation and remodeling. This contributes to increased BMD in the lumbar spine (L2–L4 segments), femoral neck, and greater trochanter in postmenopausal women.

### 7.2. Balance Ability

Balance and stability are critical biomechanical elements in dance. Dancers must maintain constant control over their center of gravity during dynamic movements, rotations, and lifts to avert falls and the subsequent risk of skeletal injuries. A substantial body of research has identified a correlation between BMD and balance proficiency. Patients afflicted with OP have been shown to demonstrate an 11% decrease in balance performance ratings compared to individuals without this condition [101]. For example, when performing one-leg balance exercises with their eyes closed or open, men aged 65–69 years rely on isometric contractions of lower limb muscles to complete movements. The muscle force generated by isometric contraction of the triceps surae acts on the attachment site of the Achilles tendon and calcaneus in a uniform manner. During this process, the mechanical stress transmitted to the calcaneus stimulates OB activity, enhancing OB function and subsequently altering the calcaneal bone mass. Consequently, balance ability in this age group of older men is significantly positively correlated with BMD [102]. A similar correlation was observed by Abdelmohsen et al. [103] between higher BMD levels and stronger physical balance in postmenopausal women. Conversely, decreased BMD has been shown to lead to increased bone fragility and reduced bone strength, further impairing postural control and physical balance. These findings suggest a dynamic interplay between BMD and balance ability, with the two factors exerting a reciprocal influence on each other’s maintenance. This collaborative relationship contributes to the enhancement of skeletal health.

**Figure 2:**
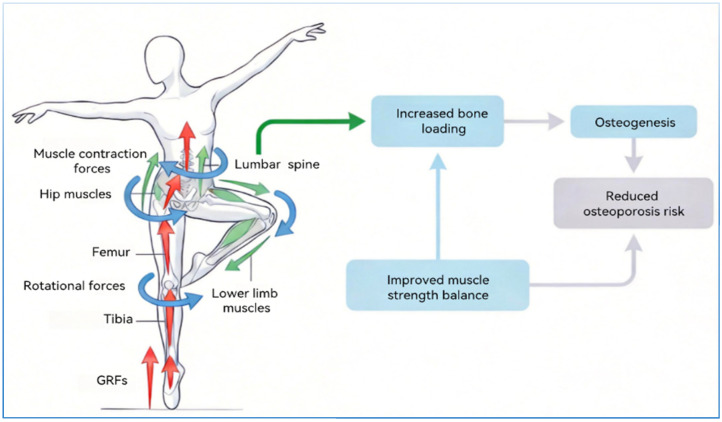
Biomechanical mechanism of dance training regulating OP. During dance training, GRFs are transmitted from the dancer’s feet through the tibia and femur to the lumbar spine. Concurrently, the dancer produces rotational forces via bodily movement. This simultaneously activates contraction of lower limb and core muscles, moderately increasing bone loading while enhancing muscular strength balance. Ultimately, this promotes bone formation and reduces the risk of OP. →: GRFs, →: muscle contraction forces, →: rotational forces.

In the domain of dance, the concept of balance assumes paramount importance. When dancers execute rapid movements or rotations, their center of gravity shifts continuously. In such instances, adjustments to parameters such as stride length, step frequency, and body posture are imperative. This enables them to sustain equilibrium while circumventing disruptions in rhythm. A substantial body of research has demonstrated that dance training significantly enhances dancers’ dynamic balance, enabling them to maintain stability throughout complex movements [10,87]. For example, the practice of traditional dance has been shown to affect alterations in gait patterns, in addition to enhancing balance capabilities [87]. The knee, which is the pivotal joint connecting the ankle and hip, frequently undergoes flexion, extension, and rotation movements of the lower limbs by dancers, and has been shown to enhance the mechanical adaptability of the patellofemoral joint. An increase in contact forces at this particular joint has been shown to extend the lever arm of the quadriceps. Furthermore, this increase has been demonstrated to enhance muscular control and precision during knee extension. This configuration has been demonstrated to enhance stability when supporting the body weight of a single leg. Moreover, it has been demonstrated that extended engagement in dance training can fortify the Achilles tendon. The enhanced mechanical properties of the Achilles tendon effectively transmit calf muscle force, thereby supporting dancers during weight transfer in movement transitions. This contributes to improved ankle stability, reducing the imbalance caused by insufficient strength and thereby preventing OP [87]. As asserted by Wu et al., low-impact dance has been demonstrated to have a significant impact on the enhancement of knee joint torque and lower limb joint range of motion in older women. This, in turn, has been shown to have a favorable impact on the reduction of the risk of falls [104] (Figure 2).

## 8. Discussion

### 8.1. The Main Findings of the Review

This review demonstrates that GRF, muscle tension, and balance-regulating stresses generated by dance movements can produce mechanical stimuli. These stimuli directly or indirectly activate multiple bone metabolism-related signaling pathways, including Wnt/β-catenin and PI3K/Akt/mTOR, thereby inhibiting OC activity and promoting OB proliferation and differentiation. Consequently, they enhance bone density and mitigate the risk of bone loss.

### 8.2. The Clinical Implications of the Review

The advantages of dance training are manifold, including its safety, accessibility, and ease of compliance. It is an effective non-pharmacological intervention for postmenopausal women, the elderly, and other populations for which long-term medication is contraindicated, addressing the high costs and significant side effects associated with drug therapies [104,105]. Moreover, dance training has been demonstrated to enhance bone metabolism, whilst concomitantly increasing muscle strength and balance. Consequently, this results in a reduction in the risk of falls amongst older adults, as well as a decrease in the incidence of osteoporotic fractures.

### 8.3. The Strengths and Limitations of This Review

This paper establishes a mechanistic framework for how dance training regulates bone metabolism through mechanical stimulation. It summarizes the biomechanical characteristics of dance and the cellular signaling pathways associated with mechanical stimulation, providing a theoretical foundation for the application of dance in preventing and treating OP.

However, the extant literature on the relationship between dance training and OP remains limited, with the majority of studies focusing on female subjects and few examining male OP patients. There is also a lack of direct mechanistic evidence and standardized protocols at the human level. Furthermore, variations in dance styles, intensity, and duration across different studies lack standardized criteria, which makes it difficult to compare intervention outcomes across different research designs. It is noteworthy that, compared to other athletic disciplines, empirical data regarding the osteogenic potential of dance remains relatively sparse. To ensure a comprehensive overview of biological mechanisms and current progress during our screening phase, we did not impose rigid inclusion criteria on dance parameters (frequency, intensity, and duration) or population characteristics (age, gender, and in vivo and in vitro models). This inclusive approach was necessitated by the limited volume of relevant literature, resulting in a degree of methodological heterogeneity across the included studies. Furthermore, the cellular signaling pathways discussed herein are largely extrapolated from general physiological principles and theoretical frameworks. Future research is required to empirically validate these specific pathways in the context of dance-induced mitigation of OP. Crucially, while existing evidence supports the osteogenic benefits of dance, direct comparative studies between dance and other weight-bearing activities—such as running or jumping, are lacking. Furthermore, future investigations should prioritize the quantification of mechanical loading profiles (e.g., GRF, loading frequency, multi-directionality, and strain variability) to elucidate the unique advantages of dance in optimizing bone development and attenuating age-related bone loss.

### 8.4. The New Direction for Future Research on the Topic

It is recommended that future research be conducted in the form of large-sample, multicenter, long-term follow-up randomized controlled trials. The study population should be expanded to include males and adolescents in order to clarify the applicability and long-term effects of dance intervention. In order to develop standardized dance intervention protocols, it is essential to quantify the efficacy of different dance types, intensities and frequencies. Concurrently, research should deepen the molecular mechanisms by which dance-induced mechanical stimulation regulates bone metabolism, paving the way for personalized dance intervention programs for OP. Furthermore, it is recommended that future studies integrate dance biomechanics with molecular biomarkers, utilizing longitudinal designs and multi-omics technologies to thoroughly investigate the effects of well-defined/standardized interventions on the organism. Special attention should be given to distinguishing the physiological effects of multidirectional impact sports, such as ballet, modern dance, and folk dance, from linear activities such as running.

## 9. Conclusions and Perspectives

In summary, dance, as a form that integrates physical activity and art, may offer a novel and promising approach for the prevention and treatment of OP. Specifically, dance training, as a form of mechanical stimulation, may regulate bone health by activating key cellular signaling pathways, including the Wnt/β-catenin, RANKL/RANK/OPG, PI3K/Akt/mTOR pathways, and so on. Nevertheless, the extant literature on the relationship between dance training and OP remains limited. To this end, future research should concentrate on strengthening the evidence base, elucidating the underlying mechanisms, and exploring optimal dance training protocols for OP intervention. This would establish dance as an integral component of comprehensive OP management.

## Data Availability

No new data were created or analyzed in this study. Data sharing is not applicable to this article.

## Abbreviations

The following abbreviations are used in this study:

OP	Osteoporosis
BMD	Bone mineral density
OC	Osteoclast
OB	Osteoblast
OCs	Osteoclasts
OBs	Osteoblasts
PGE2	Prostaglandin E2
LRP5/6	Low-density lipoprotein receptor-related protein 5/6
SOST	Sclerostin
DKK1	Dickkopf-related protein 1
OPG	Osteoprotegerin
RANKL	Receptor activator of NF-κB ligand
PI3K	Phosphoinositide 3-kinase
AKT	Protein kinase B
mTOR	Mammalian target of rapamycin
TNF-α	Tumor necrosis factor-α
Cx43	Connexin43
HC	Hemichannel
ALP	Alkaline phosphatase
Runx2	Runt-related transcription factor 2
COL-I	Collagen I
TGF-β	Transforming growth factor-β
BMP	Bone morpho-genetic proteins
Foxc1	Forkhead box C1
Max2	Msh-homebox 2
SMAD	Suppressor of mother against decapentaplegic
ERK	Extracellular signal-regulated kinase
MAPK	Mitogen-activated protein kinase
ECM	Extracellular matrix
FAK	Focal adhesion kinase
BMSCs	Bone marrow mesenchymal stromal cells
IGF-1	Insulin-like growth factor-1
GH	Growth hormone
E2	Estradiol
T	Testosterone
GRF	Ground reaction force
RFD	Rate of force development
Hh	Hedgehog
Ihh	Indian hedgehog
IGF-1	Insulin-like growth factor-1
GH	Growth hormone
E2	Estradiol
T	Testosterone
